# Tolerance of Senecavirus A to Mutations in Its Kissing-Loop or Pseudoknot Structure Computationally Predicted in 3′ Untranslated Region

**DOI:** 10.3389/fmicb.2022.889480

**Published:** 2022-05-30

**Authors:** Fuxiao Liu, Di Zhao, Ning Wang, Ziwei Li, Yaqin Dong, Shuang Liu, Feng Zhang, Jin Cui, Hailan Meng, Bo Ni, Rong Wei, Hu Shan

**Affiliations:** ^1^College of Veterinary Medicine, Qingdao Agricultural University, Qingdao, China; ^2^College of Veterinary Medicine, Inner Mongolia Agricultural University, Huhhot, China; ^3^Surveillance Laboratory of Livestock Diseases, China Animal Health and Epidemiology Center, Qingdao, China

**Keywords:** Senecavirus A, 3′ untranslated region, kissing-loop, pseudoknot, reverse genetics, mutation

## Abstract

Senecavirus A (SVA) is an emerging virus that belongs to the genus *Senecavirus* in the family *Picornaviridae*. Its genome is a positive-sense and single-stranded RNA, containing two untranslated regions (UTRs). The 68-nt-long 3′ UTR is computationally predicted to possess two higher-order RNA structures: a kissing-loop interaction and an H-type-like pseudoknot, both of which, however, cannot coexist in the 3′ UTR. In this study, we constructed 17 full-length SVA cDNA clones (cD-1 to -17): the cD-1 to -7 contained different point mutations in a kissing-loop-forming motif (KLFM); the cD-8 to -17 harbored one single or multiple point mutations in a pseudoknot-forming motif (PFM). These 17 mutated cDNA clones were independently transfected into BSR-T7/5 cells for rescuing recombinant SVAs (rSVAs), named rSVA-1 to −17, corresponding to cD-1 to −17. The results showed that the rSVA-1, -2, -3, -4, -5, -6, -7, -9, -13, and -15 were successfully rescued from their individual cDNA clones. Moreover, all mutated motifs were genetically stable during 10 viral passages *in vitro*. This study unveiled viral abilities of tolerating mutations in the computationally predicted KLFM or PFMs. It can be concluded that the putative kissing-loop structure, even if present in the 3′ UTR, is unnecessary for SVA replication. Alternatively, if the pseudoknot formation potentially occurs in the 3′ UTR, its deformation would have a lethal effect on SVA propagation.

## Introduction

Senecavirus A (SVA), also known as Seneca Valley virus (SVV), causes a vesicular disease and epidemic transient neonatal losses in swine (Oliveira et al., 2017; Bai et al., 2020). The SVA-infected case was initially found in dozens of pigs at a Canada market in 2007 (Pasma et al., 2008). However, the prototype strain of SVA (SVV-001) was originally identified as a contaminant in cell culture during cultivation of PER.C6 (transformed fetal retinoblast) cells in 2002 (Hales et al., 2008). Now, SVA has been demonstrated to be an oncolytic virus with selective tropism for some tumors with neuroendocrine characteristics (Reddy et al., 2007; Morton et al., 2010; Poirier et al., 2013). SVA is classified into the genus *Senecavirus* in the family *Picornaviridae*. To date, this virus has been the only member classified into the genus *Senecavirus*. Mature virion is a non-enveloped icosahedral particle with a diameter of approximately 27 nm (Liu et al., 2020b).

SVA is closely related to members of the genus *Cardiovirus* in genomic identity (Buckley et al., 2021). The SVA genome is a positive-sense, single-stranded, and linear RNA that, approximately 7,300 nt in length, contains one open reading frame (ORF) of polyprotein precursor, flanked by 5′ and 3′ untranslated regions (UTRs). Each SVA genome is actually a single mRNA, in which the 3′ UTR is upstream of a 3′ poly(A) tail, and the 5’ UTR has no 5′ capped structure. Alternatively, a viral protein (VPg) is covalently linked to the 5′ end of picornaviral genome (Paul and Wimmer, 2015). The polyprotein precursor can be cleaved into 12 polypeptides, namely, the L, VP4, VP2, VP3, VP1, 2A, 2B, 2C, 3A, 3B, 3C, and 3D (Liu et al., 2021a). Out of them, VP1 to VP4 are structural proteins, and the others are non-structural proteins.

Genomes of plus-strand RNA viruses can form secondary and higher-order structures, which are versatile, thus contributing not only to their stability but also to their participation in inter- and intra-molecular interactions. Those functionally important structures are referred to as *cis*-acting RNA elements, most of which are located in the highly structured UTRs, and generally involved in RNA–RNA interactions and (or) in binding key protein factors necessary for a complex process of viral propagation (Liu et al., 2009). Picornavirus-specific RNA structures, like stem-loop and pseudoknot, are commonly found in viral *cis*-acting RNA elements (Martínez-Salas, 2008; Steil and Barton, 2009). Pseudoknot is a higher-order RNA structure that contains at least two stem-loops, in which half of one stem is intercalated between the two halves of another stem. Although it has a knot-shaped three-dimensional conformation, the pseudoknot is not a true topological knot. We recently demonstrated a pseudoknot, upstream of the SVA start codon, indispensable for viral replication (Liu et al., 2021b,c), and meanwhile identified the minimum spacing between the pseudoknot and the start codon (Liu et al., 2021e).

Kissing-loop interactions, also known as loop–loop pseudoknots, are common RNA tertiary structures, resulting from base-pairing between the terminal loops of two hairpins (Bouchard and Legault, 2014). The kissing-loop structure is a special type of pseudoknot. Intra- and even inter-molecular kissing interactions are crucial for forming the complex RNA structures, which have the ability of initiating or/and regulating viral genome replication in an infected cell. Therefore, kissing-loop structures may play a role of *cis*-acting RNA element in genome replication of plus-strand RNA viruses.

Enteroviruses, as the representative picornaviruses, have been demonstrated to harbor complex RNA structures in their 3′ UTRs (Zoll et al., 2009). The typical kissing-loop structure appears to be a 6-base-paired interaction in the enteroviral 3′ UTR and is required for genome replication (Pilipenko et al., 1996; Melchers et al., 1997; Wang et al., 1999). If such an interaction is distorted by mis-pairing of each base pair involved in the higher-order RNA structure, it would cause either temperature-sensitive or lethal phenotype. The base pair constitution is more important to the existence of the tertiary structure itself than to the initiation of minus-strand RNA synthesis (Wang et al., 1999). Besides the enteroviruses, SVA (SVV-001) has also been reported to bear a putative kissing-loop structure, which potentially forms by means of two adjacent loops interacting with each other *via* a triple base-pairing pattern (Hales et al., 2008). Unfortunately, such a kissing-loop structure, albeit predicted to exist in SVV-001 3’ UTR, was not confirmed further by experiments.

The SVA CH-LX-01-2016 is a representative isolate from China (Zhao et al., 2017). It is determined to have a 668-nt 5′ UTR, and a 68-nt 3′ UTR upstream of the 3′ poly(A) tail. Its 3′ UTR is computationally predicted to possess two higher-order RNA structures: a kissing-loop interaction and an H-type-like pseudoknot, both of which, however, cannot coexist in the 3′ UTR. In the present study, we constructed a total of 17 SVA cDNA clones, out of which, seven ones harbored different mutated kissing-loop-forming motifs (KLFMs) and the others had different mutated pseudoknot-forming motifs (PFMs) in their individual 3′ UTRs. The experiment concerning virus recovery for passaging exhibited that SVA could tolerate the complete KLFM mutation, whereas its tolerance ability of PFM mutation was partially lost.

## Materials and Methods

### Prediction of Highly Structured RNAs

The SVA CH-LX-01-2016 (GenBank: KX751945.1) 3′ UTR was analyzed using the UNAFold Web Server for modeling its RNA secondary structure.^1^ The putative kissing-loop struture of SVA CH-LX-01-2016 referred to that of the SVV-001 strain (GenBank: NC_011349.1; Hales et al., 2008). A putative pseudoknot structure was predicted by the DotKnot web server.^2^

### Cell Line and Plasmid

The BSR-T7/5 cells (Buchholz et al., 1999) were cultured at 37°C with 5% CO_2_ in Dulbecco’s modified Eagle’s medium (DMEM), supplemented with 10% fetal bovine serum (VivaCell, Shanghai, China), penicillin (100 U/ml), streptomycin (100 μg/ml), amphotericin B (0.25 μg/ml) and G418 (500 μg/ml). A full-length SVA cDNA clone, tagged with the enhanced green fluorescent protein (eGFP)-encoding sequence, was constructed previously (Liu et al., 2020a). This cDNA clone, as wild-type one, was named cD-0, which would be used for rescuing the eGFP-tagged recombinant SVA (rSVA).

### Construction of Mutated SVA cDNA Clones

The cD-0 was subjected to site-directed mutagenesis by overlap extension PCR (OE-PCR) and In-Fusion® assembly for constructing 17 mutated SVA cDNA clones, cD-1 to -17. The cD-1 to -7 harbored seven types of mutated KLFMs; the cD-8 to -17 possessed 10 types of mutated PFMs. Briefly, the cD-0 was subjected to PCR separately using two pairs of primers (Table 1), forward primer 1 (FP1)/reverse primer 1 (RP1) and FP2/RP2. The first step included two independent PCRs to amplify fragment I and II from the cD-0 using the FP1/RP1 and FP2/RP2, respectively. Fragment I and II were used as templates in a tube for the second step of PCR using the FP1/RP2. The fragment I and II were fused into a longer one, followed by homologous recombination with *Bam*H I/*Pme* I-digested cD-0 using the In-Fusion® Kit (Takara, Dalian, China) to construct a mutated cDNA clone. Seventeen mutants were subjected to Sanger sequencing, purification, and agarose gel electrophoresis for confirming their mutated sites.

**Table 1:** Primers used to construct SVA cDNA clones with point mutations by overlap extension PCR.

SVA cDNA clones	FP1	RP1 (5′ to 3′)^**^	FP2 (5′ to 3′)^*^	RP2	PCR template
cD-1	#	caccgaTgcgcgttgggctatc	gatagcccaacgcgcAtcggtgctgc	※	cD-0
cD-2	#	gcaccgTagcgcgttgggctat	atagcccaacgcgctAcggtgctgcc	※	cD-0
cD-3	#	agcaccCaagcgcgttgggcta	tagcccaacgcgcttGggtgctgccg	※	cD-0
cD-4	#	gcaccgTTgcgcgttgggctatc	gatagcccaacgcgcAAcggtgctgcc	※	cD-0
cD-5	#	gcaccCaTgcgcgttgggctatc	gatagcccaacgcgcAtGggtgctgccg	※	cD-0
cD-6	#	agcaccCTagcgcgttgggctat	atagcccaacgcgctAGggtgctgccg	※	cD-0
cD-7	#	ggcagcaccCTTgcgcgttgggcta	tagcccaacgcgcAAGggtgctgccggcg	※	cD-0
cD-8	#	cccagaatcgccgCcagcaccgaagcg	cgcttcggtgctgGcggcgattctggg	※	cD-0
cD-9	#	tcccagaatcgccCCcagcaccgaagcg	cgcttcggtgctgGGggcgattctggga	※	cD-0
cD-10	#	tctcccagaatcgcGCCcagcaccgaagcgc	gcgcttcggtgctgGGCgcgattctgggaga	※	cD-0
cD-11	#	ttctcccagaatcgAGCCcagcaccgaagcgc	gcgcttcggtgctgGGCUcgattctgggagaa	※	cD-0
cD-12	#	gttctcccagaatcCAGCCcagcaccgaagcgc	gcgcttcggtgctgGGCUGgattctgggagaac	※	cD-0
cD-13	#	cccttttctgttGcgactgagttct	agaactcagtcgCaacagaaaaggg	※	cD-0
cD-14	#	cccttttctgttGGgactgagttctc	gagaactcagtcCCaacagaaaaggg	※	cD-0
cD-15	#	tcccttttctgttGGCactgagttctccc	gggagaactcagtGCCaacagaaaaggga	※	cD-0
cD-16	#	tcccttttctgttGGCCctgagttctccca	tgggagaactcagGGCCaacagaaaaggga	※	cD-0
cD-17	#	tcccttttctgttGGCCGtgagttctcccag	ctgggagaactcaCGGCCaacagaaaaggga	※	cD-0

* Mutated sites are indicated in uppercase letters.

** Reverse-complement mutated sites are also indicated in uppercase letters.

^#^5′-***ctagaccctatggatc***cccaca-3′. ^※^5′-***gctgatcagcgggttt***aaacgg-3′. Two 16-bp sequences (bold typeface) are separately designed for In-Fusion® assembly at *Bam*H I and *Pme* I sites in cD-0. FP, forward primer and RP, reverse primer.

### Rescue of rSVAs From cD-0 to -17

BSR-T7/5 cells were plated into several 24-well plates for incubation at 37°C. The cD-0 to -17 were independently transfected into cell monolayers at 70% confluency (1,000 ng/well) using Lipofectamine 2000 (Thermo Fisher, Waltham, MA, United States). The 24-well plates were cultured at 37°C, and observed under a fluorescence microscope at 72 h post-transfection (hpt), followed by one freeze and thaw cycle to collect supernatants for serial blind passages (72 h/passage) in BSR-T7/5 cells. Regardless of whether replication-competent viruses were recovered or not, the rSVAs were named rSVA-0 to -17, corresponding to cD-0 to -17.

### RT-PCR Detection

The passage-6 (P6) or − 3 (P3) progenies were harvested for extracting total RNAs, subsequently serving as templates for RT-PCR detection using the PrimeScript™ High Fidelity One Step RT-PCR Kit (Takara, Dalian, China). To detect the rSVA-0 to −12, the forward and reverse primers were FP3 (5′-CGTCCAAAACAATGACGGCTTAT-3′) and RP3 (5′-TTCCCTTTTCTGTTCCGACTGAG-3′), respectively. To detect the rSVA-13 to −17, the forward and reverse primers were FP4 (5′-AAAGTATGTGAAGAACGACAATT-3′) and RP4 (5′-AGCTTCTTCATCTCCAACTGGTA-3′), respectively. The FP3/RP3 and FP4/RP4 amplify 302- and 444-bp-long fragments, respectively. The RT-PCR underwent 45°C for 10 min, 94°C for 2 min, and then 30 cycles at 98°C (10 s), 55°C (15 s), and 68°C (5 s). Meanwhile, all samples of total RNAs were subjected to PCR detection for identifying potential plasmid residues. The PCR reaction contained 2 × PrimeSTAR Max Premix (Takara, Dalian, China) and underwent 35 cycles at 98°C (10 s), 55°C (5 s), and 72°C (5 s). The products of RT-PCR and PCR were subjected to agarose gel electrophoresis, followed by Sanger sequencing for the RT-PCR products of rSVA-1 to -12.

### 3′-Rapid Amplification of cDNA Ends (3′-RACE)

Ones of the rSVA-13- to -17, if proven to be RT-PCR-positive, would be analyzed further by 3′-RACE reaction using the HiScript-TS 5′/3′ RACE Kit (Vazyme, Nanjing, China). Briefly, total RNAs were extracted from virus-infected cells and then mixed with 3′ CDS Primer, dNTP Mix, and RNase-free H_2_O for pretreatment at 72°C up to 3 min, followed by ice bathing for 2 min. The first-strand cDNA was synthesized by that a reverse transcription system, containing the pretreated mixture, FS Buffer and Enzyme Mix, underwent one single cycle at 42°C (90 min) and 70°C (15 min). The first-strand cDNA served as template for the 3′-RACE reaction using the FP3 and Universal Primer Mix as the forward and reverse primers, respectively. The 3′-RACE products were analyzed by agarose gel electrophoresis, and then independently subcloned into linear plasmids using the TA/Blunt-Zero Cloning Kit (Vazyme, Nanjing, China) for bacterial transformation. Five single colonies were picked from each agar plate for Sanger sequencing.

### Growth Kinetics of Replication-Competent rSVAs

The rSVAs, if proven to be replication-competent, would be measured for determining their growth kinetics *in vitro*, as described previously (Liu et al., 2021c). In brief, BSR-T7/5 cells were seeded into several 12-well plates (10^6^ cells/well) for incubation at 37°C for 3 h. The rSVAs at P6 were separately inoculated (three wells/progeny, four progenies/plate, and MOI = 0.01) into cell monolayers for incubation at 37°C for 2 h, and subsequently, culture supernatants were replaced with DMEM for further incubation at 37°C. At 0, 24, 48, and 72 h post-inoculation (hpi), plates were removed from the incubator and subjected to one freeze and thaw cycle to harvest supernatants for viral titration using the Spearman–Kärber equation (Finney, 1952). Kinetic curves of virus growth were drawn using the GraphPad Prism software (Version 8.0). Data at each time point were representative of three independent experiments.

### Genetic Stabilities of Mutated Motifs During Serial Passages

All replication-competent rSVAs underwent 10 serial passages in BSR-T7/5 cells. The P10 progenies were detected by RT-PCR using the FP3/RP3 or by 3′-RACE reaction, followed by Sanger sequencing for revealing genetic stabilities of mutation motifs.

## Results

### SVA 3′ UTR Harbors a Kissing-Loop or Pseudoknot Structure

**Figure 1:**
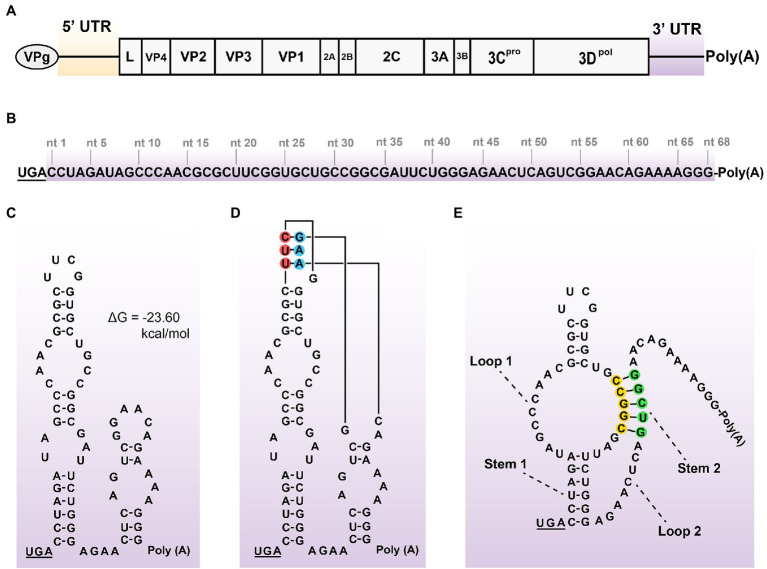
Schematic representations of SVA genome, 3’ UTR sequence, and its potential structures. Positive-sense, single-stranded, and linear SVA genome **(A)**. A typical genome is composed of 5’ UTR, polyprotein ORF, 3’ UTR, and poly(A) tail. The VPg is covalently linked to the 5′ end of genome. The proportion of elements does not exactly match them in the genome. 68-nt-long sequence of 3’ UTR **(B)**. Putative secondary structure of 3′ UTR **(C)**. The 3′-UTR sequence is analyzed using the UNAFold Web Server (http://www.unafold.org/) for modeling its RNA secondary structure, which contains two adjacent stem-loops. The stop codon (UGA) is underlined. Two terminal loops are predicted to have a potential “kissing interaction” with each other by means of a triple base-pairing pattern (**D**, red/blue circle-marked). Such an interaction refers to that of the SVV-001 strain (Hales et al., 2008). H-type-like pseudoknot **(E)**, composed of two stems (Stem 1 and 2) and two loops (Loop 1 and 2), is predicted in 3’ UTR by the DotKnot web server. The Stem 2 contains two PFMs, separately marked with yellow and green circles.

The genome of SVA CH-LX-01-2016 was schematically shown in Figure 1A. Its 3′ UTR was composed of 68 nucleotides (Figure 1B), not including the stop codon (UGA) and poly(A) sequence. The 68-nt-long RNA sequence was predicted to form one secondary structure (ΔG = −23.60 kcal/mol) that contained two stem-loops adjacent to each other (Figure 1C) using the UNAFold Web Server. The tertiary structure of kissing-loop was previously predicted to be present in the 3′ UTR by means of a triple base-pairing pattern (U-A, U-A, and C-G, Figure 1D; Hales et al., 2008). Alternatively, an H-type-like pseudoknot (Figure 1E) was computationally predicted in the 3′ UTR using the DotKnot web server. This pseudoknot was composed of two stems (Stem 1 and 2) and two loops (Loop 1 and 2). Because the Loop 1 was an internal loop, rather than a terminal loop, such a pseudoknot was called H-type-like pseudoknot here. The Stem 2 contained two PFMs, separately marked with yellow and green circles in Figure 1E. The kissing-loop and the pseudoknot could not coexist in the 3′ UTR.

### Seventeen Mutated cDNA Clones Are Constructed

**Figure 2:**
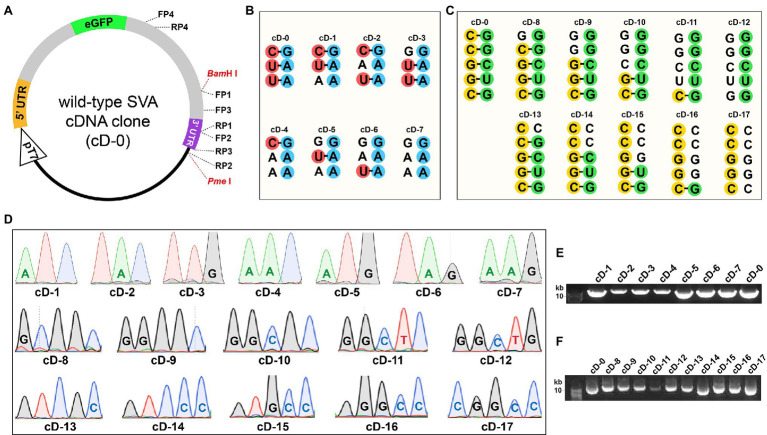
Construction of 17 full-length SVA cDNA clones. Schematic representation of eGFP-tagged SVA cDNA clone without mutation **(A)**. Dotted lines indicate FP1/RP1-, FP2/RP2-, FP3/RP3-, FP4/RP4-, and *Bam*H I/*Pme* I-targeted sites. The proportion of elements does not exactly match them in the cD-0 plasmid. Wild-type and mutated KLFMs **(B)**. Mutated sites are unmarked with red/blue circles. Wild-type and mutated PFMs **(C)**. Mutated sites are unmarked with yellow/green circles. Sanger sequencing chromatograms of 17 cDNA clones **(D)**. Mutated sites are marked with letters inside their sequencing peaks. Agarose gel electrophoresis of cD-1 to −17 after plasmid purification **(E**,**F)**.

The eGFP-tagged SVA cDNA clone (cD-0) was a wild-type plasmid, schematically shown in Figure 2A, in which FP1-, RP1-, FP2-, RP2-, FP3-, RP3-, FP4-, RP4-, *Bam*H I-, and *Pme* I-targeted sites were indicated with dotted lines. The cD-0 was genetically modified to construct 17 mutants (cD-1 to -17) using OE-PCR and In-Fusion® assembly. Mutants’ motifs were shown in Figures 2B,C, in which mutated sites were not labeled with colorful circles. The 17 mutants were approximately 13.4 kb in length. They were subjected to Sanger sequencing for confirming mutated sites in their individual motifs (Figure 2D), and then to agarose gel electrophoresis (Figures 2E,F).

### Ten Replication-Competent rSVAs Are Rescued From cDNA Clones

**Figure 3:**
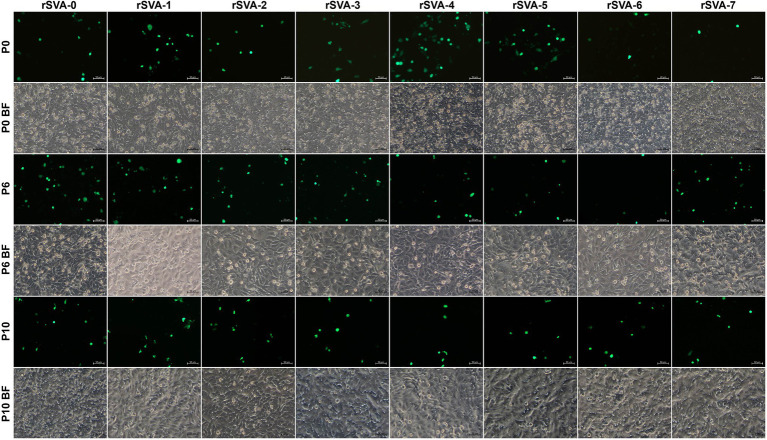
Profiles of rSVA-0- to −7-expressed eGFPs in BSR-T7/5 cells at P0, P6, and P10. Cell monolayers are separately transfected with cD-0 to −7 and subjected to one freeze and thaw cycle at 72 hpt (P0) to collect supernatants for 10 serial passages. BF, bright field.

**Figure 4:**
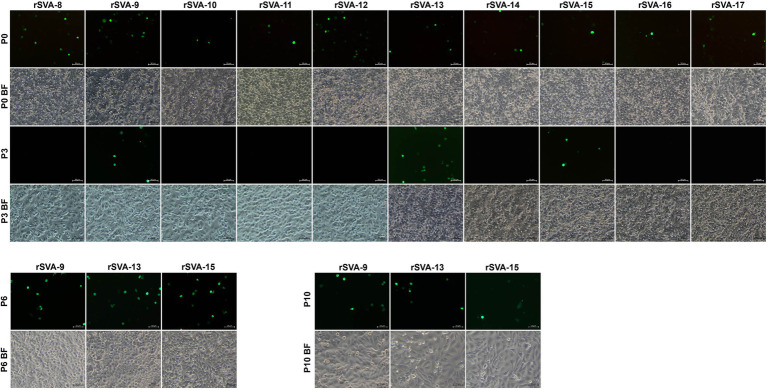
Profiles of rSVA-8- to −17-expressed eGFPs in BSR-T7/5 cells during passaging. Cell monolayers are separately transfected with cD-8 to −17. At 72 hpt (P0), cell cultures undergo one freeze and thaw cycle to collect supernatants for three serial passages. Three eGFP-expressing groups (rSVA-9, −13, and −15) are consecutively passaged in cells until P10. BF, bright field.

**Figure 5:**
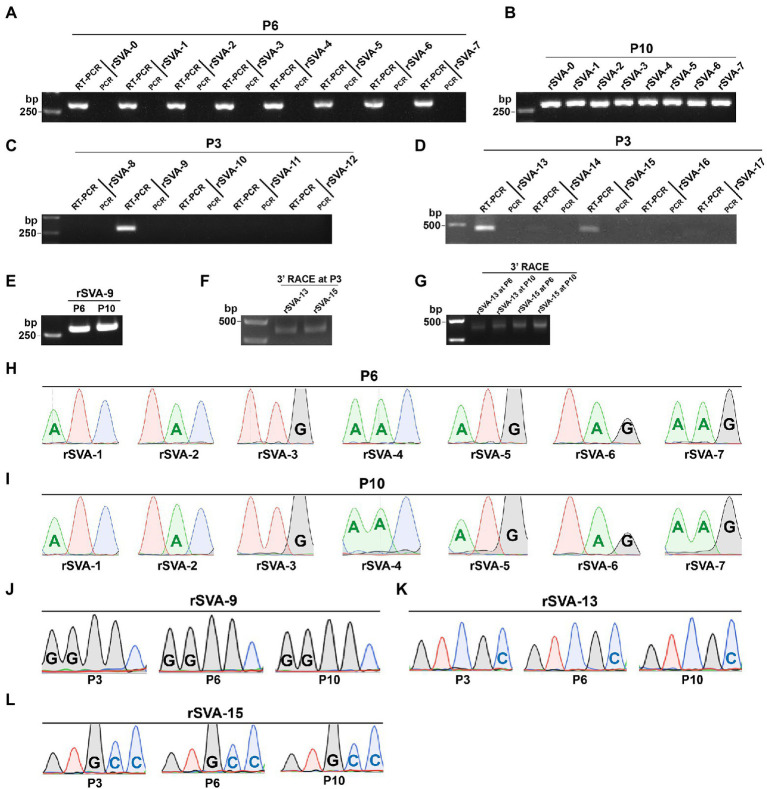
Identification of replication-competent rSVAs rescued from their individual cDNA clones. RT-PCR detection of rSVA-0 to −7 at P6 **(A)** and P10 **(B)**. PCR analysis is simultaneously performed to demonstrate no interference of plasmid residues, the same below. RT-PCR detection of rSVA-8 to −12 **(C)** and −13 to −17 **(D)** at P3. RT-PCR detection of rSVA-9 at P6 and P10 **(E)**. 3’-RACE reaction of rSVA-13 and -15 at P3, P6, and P10 **(F**,**G)**. Sanger sequencing chromatograms of RT-PCR products from rSVA-1 to −7 samples at P6 **(H)** and P10 **(I)**. Sanger sequencing chromatograms of RT-PCR products from three rSVA-9 samples **(J)**. Sanger sequencing chromatograms of 3’-RACE reaction products from rSVA-13 **(K)** and −15 **(L)** samples.

The cD-0 to -17 were separately transfected into BSR-T7/5 cells for rescuing recombinant viruses. Green fluorescence was observable on all cell monolayers transfected with mutants at 72 hpt (Figures 3, 4, Panel P0). A total of 11 groups, rSVA-0, -1, -2, -3, -4, -5, -6, -7, -9, -13, and -15, always had the fluorescence-emitted phenotype during blind passaging (Figures 3, 4, Panel P3, P6 and P10). All 17 groups were analyzed by the one-step RT-PCR after serial viral passaging, confirming that these 11 rSVAs were successfully rescued from their individual cDNA clones (Figures 5A,C,D, Lane RT-PCR). As a control, PCR analysis indicated no cDNA clone contamination affecting RT-PCR detection (Figures 5A,C,D, Lane PCR). The rSVA-13 and -15 were additionally subjected to 3′-RACE reaction at P3 (Figure 5F). RT-PCR and 3′-RACE products were subjected to Sanger sequencing, which indicated that none of the progenies underwent sequence variation (Figures 5H: P6; 5J: P3; 5K: P3; 5L: P3).

### Mutated rSVAs Have Similar Growth Kinetics to That of the rSVA-0

**Figure 6:**
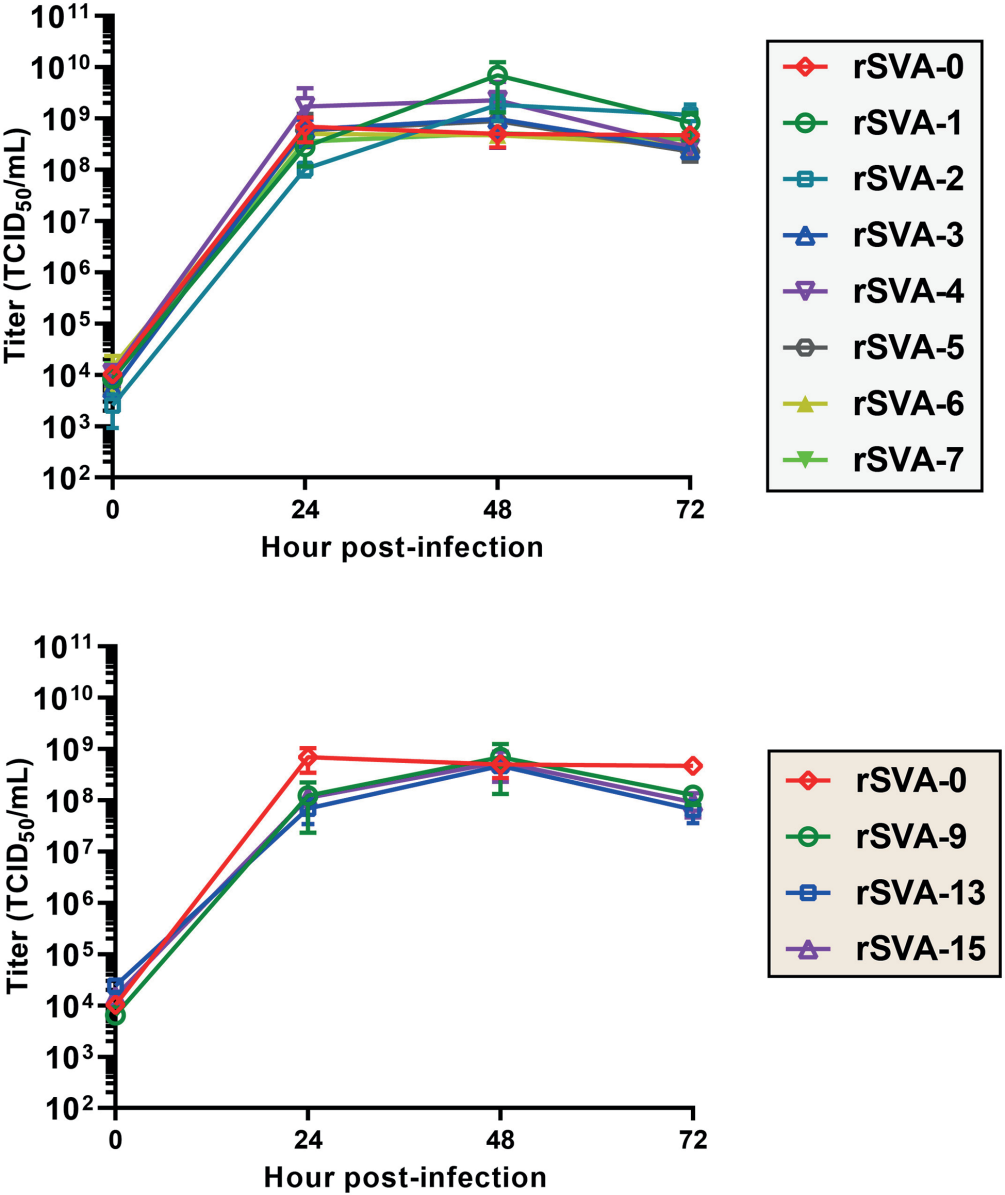
Multistep growth curves of replication-competent rSVAs at P6. Viral titers are measured using the Spearman–Kärber equation. Data at 0, 24, 48, and 72 hpi are representative of three independent experiments. Error bar indicates standard deviation.

To compare growth kinetics of mutated rSVAs with that of the rSVA-0 *in vitro*, BSR-T7/5 cell monolayers were inoculated with viral progenies at MOI of 0.01. Viral titers were measured at 0, 24, 48, and 72 hpi. Multistep growth curves were drawn (Figure 6). The result showed that these 10 mutated rSVAs had similar growth kinetics to that of the rSVA-0, suggesting none of the mutated motifs exerting a significant impact on viral replication.

### All Mutated Motifs Are Genetically Stable During 10 Passages

At P6 or (and) P10, all virus-infected cultures were collected for RT-PCR or 3′-RACE reaction. Their products showed single bands of expected sizes on agarose gels (Figures 5B,E,G). Sanger sequencing displayed all mutated motifs that were genetically stable at P6 or (and) P10 (Figures 5I: P10; 5J: P6 and P10; 5K: P6 and P10; 5L: P6 and P10).

## Discussion

SVA is an emerging virus that can induce vesicular and/or ulcerative lesions on pigs’ snouts, oral mucosae, coronary bands as well as hooves, and also cause epidemic transient neonatal losses in pigs. This virus has affected pig farms in some countries, such as the United States (Singh et al., 2012), Thailand (Vannucci et al., 2015), and China (Wu et al., 2017). Due to SVA being an emerging pathogen, much remains to be elucidated about its molecular mechanisms in pathogenicity, immunity, replication, and so forth. Reverse genetics is an ideal tool to recover genetically modified SVAs for exploring these molecular mechanisms. We previously established an efficient platform of reverse genetics to rescue the wild-type (Liu et al., 2021a) or reporter-tagged (Liu et al., 2020a, 2021d) SVA. This reverse genetics platform was recently used for uncovering many molecular mechanisms in an RNA pseudoknot and its adjacent motif upstream of the viral start codon (Liu et al., 2021b,c,e).

Picornaviral 3′ UTR is generally a highly structured region. Both 3′ UTR and poly(A) tail are required for efficiently initiating enteroviruses, e.g., poliovirus and Coxsackievirus, to synthesize their own minus-strand RNAs (Rohll et al., 1995; Melchers et al., 1997; Mirmomeni et al., 1997; Wang et al., 1999). Structural analysis indicated that the enteroviral 3′ UTR contained two or three hairpin structures, named domain X, Y, and Z. A kissing-loop structure exists between domain X and Y *via* a six base-pairing pattern. The kissing domain K is stacked to the helix of domain X for generating one coaxial helical domain, which, connected to the domain Y or to the stacked coaxial helical domain Y-Z, forms the overall structure of replication origin to initiate minus-strand RNA synthesis (Pilipenko et al., 1996; Melchers et al., 1997; Mirmomeni et al., 1997). If mutations disrupt the formation of kissing interaction, enteroviral RNA synthesis would be inhibited. Except those of enteroviruses, kissing-loop structures of other picornaviruses have been poorly defined as yet.

Hales et al. (2008) predicted a kissing-loop structure in the SVV-001 3′ UTR (Hales et al., 2008). Unfortunately, it has not been demonstrated whether such a putative structure is able to exert an impact on viral replication in cells. This prompted us to conduct the present study for clarifying this issue. At first, we used the UNAFold web server, formerly known as Mfold web server (Zuker, 2003), to predict the RNA secondary structure of SVA CH-LX-01-2016 3′ UTR (Figure 1C), proven to be extremely similar to that of the SVV-001. Subsequently, a set of mutated eGFP-tagged cDNA clones were constructed for testing whether mutated KLFMs could interfere with recovery of rSVAs. The eGFP facilitates real-time observation of plasmid-transfected or virus-inoculated cells. Green fluorescence, if emitted from a cell monolayer, implies cells infected with a given rSVA.

We proposed here a preliminary hypothesis that if the putative kissing-loop structure definitely existed in the 3′ UTR and moreover played a key role in viral replication, at least one rSVA could not be rescued from the cD-1 to -7. Nevertheless, all seven recombinants, rSVA-1 to -7, were successfully rescued from their individual cDNA clones. The rSVA-1- to -7-infected cell monolayers not only emitted green fluorescence, but also showed a similar proportion of fluorescent cells at each passage (Figure 3). We recently demonstrated that a given eGFP-tagged SVA, albeit rescued from a cDNA clone with mutated pseudoknot motif in the 5′ UTR, reverted back to its own wild-type genotype with viral passaging (Liu et al., 2021c). Thus, it was necessary in the present study to use Sanger sequencing for revealing genetic stabilities of all mutated KLFMs. The result exhibited rSVA-1 to -7 with high-fidelity characteristics in their mutated KLFMs at P6 (Figure 5H) and P10 (Figure 5I). Moreover, these seven recombinants showed their growth kinetics similar to that of the rSVA-0 (Figure 6, Upper panel).

The above-mentioned results suggested that the kissing-loop structure might not be present in the 3′ UTR of wild-type SVA, or even if present, was unnecessary for viral propagation. Based on this conclusion, we assume other key *cis*-acting RNA elements that exist in the 3′ UTR and can affect viral characteristics, like antigenome replication and virion packaging. The reason why such an assumption was proposed here was that hepatitis A virus (HAV) had been reported to possess two alternative models of RNA structure in its 3′ UTR. HAV also belongs to the family *Picornaviridae*. The first model contains a “kissing” interaction, and the second one represents an H-type pseudoknot, both of which may clarify higher-order RNA structures within the HAV 3′ UTR (Dollenmaier and Weitz, 2003).

Owing to the kissing-loop structure unnecessary for SVA, it remains to be elucidated whether other crucial RNA elements exist in the viral 3′ UTR. The DotKnot is an efficient algorithm, able of finding pseudoknots with high accuracy (Sperschneider and Datta, 2010). Thus, the DotKnot web server was used to remodel the SVA 3′ UTR for predicting other higher-order RNA structures within it. An alternative one, H-type-like pseudoknot (Figure 1E), was predicted to exist potentially in the 3′ UTR. In an effort to clarify whether the putative pseudoknot formation was required for SVA replication, 10 extra SVA cDNA clones were constructed for rescuing recombinants with mutated PFMs. As a result, only rSVA-9, -13, and -15 could be recovered. The rSVA-9 had two consecutive point mutations in a PFM. The rSVA-13 and -15 had one single and three consecutive point mutations in the other PFM, respectively. Interestingly, these two mutated PFMs did not complement each other, suggesting other potential elements or factors possibly involved in SVA replication.

Picornaviral 3′ UTR is believed to be a major *cis*-acting RNA element for regulating antigenome synthesis. Different picornaviruses generally contain diverse sequences of 3′ UTR, which form various RNA secondary or higher-order structures (Cui and Porter, 1995; Wang et al., 1999; Dollenmaier and Weitz, 2003; Serrano et al., 2006). Diverse RNA structures would function in different ways to regulate viral propagation (Liu et al., 2009). Interestingly, some picornaviruses could tolerate the complete deletion of 3′ UTR sequence from their genomes (Todd et al., 1997). As an emerging virus, SVA has not been widely explored regarding its molecular mechanism as yet. In the present study, we demonstrated that the putative kissing-loop structure (Figure 1D), even if present in the SVA 3′ UTR, was unnecessary for virus recovery and passaging. Alternatively, the putative pseudoknot, if subjected to one single or multiple point mutations in a PFM, would confer a lethal effect on virus recovery. Neither the kissing-loop nor the pseudoknot was structurally confirmed further by enzymatic and chemical probing here, whereas the present study unveiled viral abilities of tolerating point mutations in the putative KLFM or PFMs.

## Data Availability Statement

The original contributions presented in the study are included in the article/supplementary material, and further inquiries can be directed to the corresponding authors.

## Author Contributions

FL conducted the experiments and wrote the manuscript. DZ, NW, ZL, YD, SL, FZ, JC, and HM performed the experimental works and finished the data analysis. BN, RW, and HS provided the funding. All authors contributed to the article and approved the submitted version..

## Funding

This work was supported by the Expert Fund from the Shandong Technique System of Pig Industry for Disease Control (SDAIT-08-07) and the Innovation Fund, funded by China Animal Health and Epidemiology Center.

## Conflict of Interest

The authors declare that the research was conducted in the absence of any commercial or financial relationships that could be construed as a potential conflict of interest.

## Publisher’s Note

All claims expressed in this article are solely those of the authors and do not necessarily represent those of their affiliated organizations, or those of the publisher, the editors and the reviewers. Any product that may be evaluated in this article, or claim that may be made by its manufacturer, is not guaranteed or endorsed by the publisher.

## Footnotes

^1^ http://www.unafold.org/

^2^ https://dotknot.csse.uwa.edu.au
